# Implementing public health vending machines on the reservation lands of the Bois Forte Band of Chippewa (United States)

**DOI:** 10.1186/s12954-025-01244-6

**Published:** 2025-05-28

**Authors:** Sean T. Allen, Kristin E. Schneider, Maisie Conrad, Pam Hughes, Allison O’Rourke, Molly C. Reid, Cathy Chavers, Christopher G. Kemp, Melissa Walls

**Affiliations:** 1 Department of Health, Behavior and Society, Johns Hopkins Bloomberg School of Public Health, Johns Hopkins University, 624 N. Broadway, Hampton House 184, Baltimore, MD 21205 USA; 2 Department of International Health, Johns Hopkins Bloomberg School of Public Health, 415 N Wolfe St, Baltimore, MD 21205 USA; 3 Department of Pharmacy Practice and Pharmaceutical Sciences, University of Minnesota– College of Pharmacy, Duluth, 55812 USA; 4 Department of Mental Health, Johns Hopkins Bloomberg School of Public Health, 624 N. Broadway, Baltimore, MD 21205 USA; 5 Bois Forte Band of Chippewa, 5344 Lake Shore Drive, Nett Lake, MN 55772 USA

**Keywords:** Public health vending machines, Harm reduction, American Indian, Indigenous, People who use drugs

## Abstract

**Background:**

Public health vending machines (PHVMs) have been shown to improve access to numerous health-promoting supplies. However, they are understudied in the United States and no study of which we are aware has examined their implementation on American Indian reservation lands.

**Objective:**

This study describes how PHVMs were implemented on the Bois Forte Band of Chippewa Reservation lands in Minnesota (United States) and associated costs during the first 24 weeks of operations.

**Methods:**

We summarize the steps undertaken to implement PHVMs on reservation lands. We describe formative research in which we assessed willingness to use a PHVM among a sample of people who use drugs. Then, we describe how we worked with community members to ensure the appropriateness of a PHVM initiative. Finally, we present utilization data from the first 24 weeks of PHVM operations and describe associated implementation costs.

**Results:**

In May 2022, the academic research team presented to the Bois Forte Reservation Tribal Council about PHVMs and requested a Resolution that authorized their implementation on Reservation lands. The Resolution was unanimously approved, and the team began working with a local Community Research Council to determine the most appropriate way to implement the PHVMs. Prior to implementation, we conducted a survey among people who used drugs (*N* = 227) on Reservation lands and found that most (56%) reported willingness to use PHVMs if they were available. In October 2023, two PHVMs were installed. During the first 24 weeks of operations, there were 2,534 successful dispensations at the PHVMs. Items dispensed through the PHVMs included COVID-19 test kits, naloxone, drug test strip kits, menstrual supply kits, wound care kits, sterile injection equipment kits, pregnancy tests, safer sex kits, and HIV self-test kits. In total, the PHVM initiative cost approximately $30,406.75 (USD) during its initial 24-week implementation period.

**Conclusions:**

The Bois Forte Band of Chippewa took swift action upon learning about the potential public health benefits of PHVMs and immediately authorized their implementation, catalyzing a collaborative effort between local community members and academic partners. Thousands of life-sustaining supplies were dispensed at the PHVMs, supporting the prevention and treatment needs of the community.

## Background

American Indian/Native American (hereafter, “Indigenous”) Peoples in the United States (US) experience disproportionate burdens of substance use-related adverse health outcomes compared to their non-Native counterparts [1–4]. For instance, in 2020 and 2021, age-adjusted overdose fatality rates were highest among American Indian/Alaska Natives [5]. These recent inequities in overdose fatality rates are not an isolated occurrence, but rather part of a larger trend as opioid-related mortality among Indigenous Peoples increased from 1999 to 2019 [6]. For many Indigenous communities in the US, the addiction and overdose crisis can be linked to historical trauma, colonization, and structural racism [7–9]. Further, the US government has a well-documented history of failing to honor treaties with Indigenous Peoples, resulting in the loss of Indigenous Knowledge, ancestral lands, and culture [7–9]. Taken together, these events and experiences culminate in many Indigenous communities having inadequate resources to respond effectively to rapid escalations in substance use and associated harms.

In response to the addiction and overdose crisis, many Indigenous communities are embracing harm reduction interventions to prevent substance use harms [10–12]. Broadly, harm reduction interventions aim to minimize the harms associated with substance use without requiring abstinence [12–14]. Although there are many harm reduction interventions, syringe services programs are one of the most well-known [15]. These programs provide people who inject drugs with access to sterile injection equipment and a myriad of other services and resources, such as vaccinations, referrals to substance use treatment, and wound care [15]. Syringe services programs have been studied extensively throughout the world; their implementation has been shown to decrease community level HIV transmission and provide life-saving services to people who inject drugs [15–17]. There are several noteworthy examples of Indigenous communities launching syringe services programs; for example, the Gwayakobimaadiziwin Bad River Harm Reduction program has operated since 2015 on the Bad River Band of Lake Superior Chippewa Reservation [11]. Initiatives aimed at distributing the overdose reversal medication naloxone are another example of harm reduction [18–23]. By increasing the availability of naloxone, particularly among people who use drugs and their network affiliates, overdose fatalities may decrease at the community level [18–21]. There are many examples of Indigenous-led naloxone distribution programs throughout the United States. For example, the syringe services program at the Indigenous Peoples Task Force in Minnesota has offered an array of harm reduction services, including naloxone access, for several decades [24]. Although the scale of harm reduction interventions in Indigenous communities is increasing, their implementation remains challenging given inadequate funding and the sprawling rural geographies of many reservation lands.

Implementing public health vending machines (PHVMs) is an evidence-based harm reduction strategy that holds considerable potential to improve public health in Indigenous communities [25, 26]. PHVMs are similar to snack vending machines, but dispense free public health supplies, such as wound care kits, sterile injection equipment, naloxone, pregnancy tests, condoms, and flashlights [25, 27–30]. PHVMs have operated outside the US for decades and their implementation has been well-studied [27]. For example, PHVM implementation has proven effective for reaching people who use drugs that do not access services at syringe services programs (e.g., younger people who inject drugs) [28]. In the US, PHVMs are a relatively new intervention. They were first implemented in 2017 at a syringe services program in Clark County, Nevada, where they primarily distributed sterile injection equipment and naloxone [26]. According to a 2022 study, naloxone dispensation at PHVMs in Clark County was associated with significant reductions in overdose mortality [26]. 

Although existing literature about PHVM implementation is informative, no studies of which we are aware have been conducted to understand their acceptability among people who use drugs on American Indian reservation lands. This represents a noteworthy gap in the scientific literature given the scale of addiction and overdose inequities among Indigenous Peoples [1–3]. Further, PHVM implementation may offer Indigenous communities low-barrier access to an array of essential health supplies, particularly in areas that are geographically isolated (e.g., some reservation lands) and lack adequate funding to support robust harm reduction services. Scaling up access to PHVMs on American Indian reservation lands also requires funding; however, there are very few operational PHVMs in Indian Country and no published examinations of their associated costs.

To address these gaps, this research summarizes the steps our team undertook in collaboration with the Bois Forte Band of Chippewa in the Northern Midwest US to implement two PHVMs on reservation lands [31–33]. We describe formative research in which we assessed hypothetical community-level willingness to use a PHVM if a machine were available among a sample of people who use drugs on reservation lands. Next, we describe the process of working with community members and leaders to ensure the appropriateness of a planned PHVM program, as well as practical considerations that contributed to the feasibility and uptake of implementation. Then, we present utilization data from the first 24 weeks of PHVM operations to characterize intervention penetration to community members. We also estimated implementation costs.

## Methods

### Study context & community-based participatory research (CBPR) approach

This project occurred in collaboration with the Bois Forte Band of Chippewa (also referred to as Ojibwe) in Northern Minnesota (US). The Bois Forte Reservation is located about 45 miles south of the Canadian border [34]. According to 2020 census data, there are slightly less than 1,000 residents on the Bois Forte Reservation and off-Reservation Trust Land [35]. Many members of the Bois Forte Band of Chippewa migrate cyclically on and off Reservation lands. The Bois Forte people are resilient and well-known for their traditions, including harvesting wild rice, tapping maple trees, and picking berries [34]. The Bois Forte Band of Chippewa also operates a popular casino on Lake Vermilion that offers gaming, events/concerts, and outdoor recreational activities [34]. The senior author (MW) had personal, cultural, and longstanding partnerships with the Bois Forte community and is an Indigenous researcher. Understanding that Bois Forte wanted additional data about the addiction and overdose crisis on their reservation, the senior author worked with the first author and a team of investigators and community partners, to develop a project that aimed to generate action-oriented data to mitigate substance use harms. The project included surveying local people who use drugs about their substance use and service needs as well as willingness to utilize services (e.g., PHVMs) if they were available. A derivative product of this project also involved disseminating information about public health interventions that were available in other communities to relevant constituencies at the Tribe (e.g., Reservation Tribal Council, healthcare providers).

Our team approaches this work following principles of Community and Tribally Based Participatory Research (CBPR/TBPR), which offer principles and strategies to address power imbalances in research partnerships, engage community and university teams in co-leadership roles, and work together on a given topical area for the purposes of change and impact [36, 37]. Our approach is further rooted in respect and affirmation of Tribal sovereignty with an intentional shift away from transactional research towards relational approaches that honor Tribes’ sovereign rights to govern research within their own lands [38]. In this case, early discussions among authors led to a shared decision to approach the Reservation Tribal Council to present our plans for collaborative research and action in an open Council meeting. We explicitly requested permission to move forward with the establishment of a Community Research Council (CRC) to co-lead the project and to write grants to fund project activities. We received a formal Resolution to support this work (Resolution No. 89-2020) prior to applying for grant funding. Once funded, we spent the first several months engaged in relationship building to recruit and create a CRC comprised of Tribal members with lived experience relevant to substance use research and/or who occupied professional roles to support people who use drugs. Our initial activities with the CRC centered around collaborative meetings, shared value-setting, and co-learning activities and discussions to build and maintain trust and a sense of shared purpose and understanding critical to and ethically necessary in American Indian health research [39, 40]. As we moved forward, CRC and university team members collaborated on project methods, decisions for appropriate use and interpretation of data, and steps for action, including seeking appropriate permissions and managing logistical steps necessary for vending machine implementation. One CRC member and a former Tribal Leader serve as co-authors on this manuscript. These efforts all aim to center community members as core actors and beneficiaries of research that directly addresses local priorities and needs [40, 41]. Data and products resulting from our collaboration are returned and belong to the Tribe in keeping with data sovereignty principles.

### Implementation process

We summarize the process of working collaboratively with the Bois Forte Band of Chippewa to implement PHVMs. We describe how we initially approached the Reservation Tribal Council to gain approval to implement PHVMs. We also describe our work to ensure implementation appropriateness in terms of culture and community perceptions of the supplies being distributed.

### Community-level willingness

Data pertaining to community-level willingness to use a PHVM were collected as part of the “Our Stories Matter” study conducted on the reservation lands of the Bois Forte Band of Chippewa. Surveys were collected in September and October 2022 at a local Powwow, a tribally operated casino, a housing facility, and in a public space adjacent to a residential area on the reservation. To be eligible, participants had to be at least 18 years old and have used any drug in their life. Surveys were administered via audio computer-assisted self-interview (ACASI) and took about 15 min to complete. Participants received either a $20 Amazon or Visa gift card for participating. All study activities were conducted in line with a CBPR orientation, which we used to ensure activities were informed by local expertise and aligned with cultural values and practices. Throughout the study, the CRC was an equal partner in developing the study design, survey measures, and implementation approach. The CRC was also consulted about what items should be stocked in the PHVMs. This project was also reviewed and approved by the Institutional Review Board at the Johns Hopkins Bloomberg School of Public Health.

### Survey measures

Willingness to use a PHVM was captured by the following survey item, “Would you be willing to use a vending machine to get things like sterile injection equipment or the overdose reversal medication naloxone for free if it were available in your community?” (yes/no). Sociodemographic characteristics included age (in years), gender (man, woman, other), sexual orientation (categorized as a binary variable indicating sexual minority status), education level (less than high school, high school equivalent/GED, some college or more), self-identified current homelessness (yes/no), current health insurance (yes/no), current relationship status (single/in a relationship), current employment (not working, working full time, working part time), and having gone to bed hungry due to a lack of food in the past 6 months (yes/no). Participants also reported their race and ethnicity. Participants reported how many fatal and non-fatal overdoses they had witnessed in the past 6 months. From these counts, we created two binary variables to indicate witnessing any fatal or non-fatal overdoses in the past 6 months. We also included several substance use measures, including a binary indicator for if the participant had reported using drugs other than cannabis in the past 6 months. Among those who reported any drug use in the past 6 months, we created binary indicators for any use of each route of administration (smoking, snorting, swallowing, injecting) and any non-medical use of opioids (heroin, fentanyl, prescription opioids), stimulants (cocaine, methamphetamine, prescription stimulants), and other drugs. Importance of Indigenous spirituality was assessed with the question: “How important are traditional Indigenous spiritual values to the way you lead your life?” and responses were categorized as very important/somewhat important and not too important/not at all important. Cultural identity was measured via an adapted 7-item scale that included questions about pride in Indigenous identities and connections to other Indigenous Peoples [42]. Responses were measured on a four-point Likert scale (3- strongly agree, 2 - agree, 1- disagree, 0 - strongly disagree) and summed for a total score (possible range: 0 to 21).

### Survey analyses

In total, *N* = 336 unique surveys were collected and *n* = 227 reflected persons who self-identified as Indigenous. Given our focus on PHVM implementation in an Indigenous context, we limited our analyses to the *N* = 227 surveys that indicated persons identified as Indigenous. We estimated the prevalence or mean of each sociodemographic, cultural, and substance use measure among the 227 participants to characterize the sample. We then used chi square and t-tests, as appropriate, to assess the association of each other variable with willingness to use a PHVM. In cases of very small cell sizes, we used Fisher’s exact tests. Analyses were conducted using Stata 18.

### PHVM dispensing data

We summarized the dispensing data from the first 24 weeks of operations (October 2023 to March 2024) of the two PHVMs to characterize penetration of the intervention in the community. We selected this time period in response to community requests for a clearer understanding of the operational costs associated with PHVM implementation. Throughout the implementation process, we also received similar inquiries from other communities seeking insight into these costs. The vending machine software captured the exact date and time of each transaction, as well as what item was dispensed. Based on discussions with local community members, including the CRC, the PHVMs were stocked with items of public health relevance; for example, the machines featured items pertaining to oral hygiene, overdose prevention, food security, infectious disease prevention and testing, basic needs (e.g., clothing), first aid, diabetes, and sexual health. More specifically, the vending machines dispensed: granola and protein bars, over-the-counter (OTC) pain relievers, flashlights, dental kits (toothbrush and toothpaste), COVID-19 rapid tests, men’s and women’s underwear, nasal naloxone kits, drug test strip kits (i.e., fentanyl and xylazine test strips), hat/gloves, menstrual supplies, sunglasses, wound care kits, bandages, socks, sharps containers, lip balm, glucose tablets, sterile injection equipment kits (2 syringes, 2 alcohol wipes, 1 cooker kit), hand sanitizer, pregnancy tests/condom kit, protein drinks, bottles of water, face masks, HIV self-test kits, sterile cooker kits (1 cooker, 1 filter), and sunscreen sachets. Cooker kits were only briefly dispensed alone and were combined with the sterile injection equipment kits for most of the operational period. This change was made due to a lack of visual clarity of the cooker kits when alone (i.e., people could not tell what they were). We included the small number of cooker kits dispensed before this change was made for completeness. We summarized the number of each item type that was dispensed, overall and by PHVM location. We then summarized the day and time data of each transaction to provide percentages of transactions that occurred each day of the week and in each 2-hour period of a day, overall and by location.

### Cost estimation

The cost estimation was done with the goal of producing estimates that can help inform Tribes or other entities interested in implementing a similar PHVM strategy about the approximate expenses they can expect to incur. As such, we assumed the healthcare perspective for cost estimation to inform future planners of PHVM initiatives on programmatic costs. Further, the PHVM initiative was supported by a combination of community members, venues, and agencies. We estimated the material costs of operating the PHVMs for the first 24 weeks (i.e., six months). We compiled information from all purchase orders associated with the PHVMs. We first calculated start-up implementation costs reflecting PHVM purchase, freight charges, technology fees, and machine graphics (i.e., artwork wrapped around the PHVMs). We then calculated recurrent costs associated with machine stock based on the average price per unit for each item type, as prices fluctuated over the operation period and by vendors. Using the average price per unit, we calculated the estimated average cost by item by multiplying the number of a given item dispensed by the average price per unit (in USD). We then created an upper and lower bound for costs based on the variation in price per item. We calculated the lower bound using the lowest price that was paid per unit multiplied by the number of items dispensed and the upper bound by using the highest price paid per unit. The pricing of some items did not vary over the study period, so these items do not have meaningful upper and lower bounds. We then estimated the total recurrent stock costs for the first 24-week period by summing the average item costs. Next, we developed projections for the total recurrent costs over one year by extrapolating from 24 weeks of empirical cost estimates. Finally, we calculated the total projected investment in PHVM operations during the first year of operations by combining total projected recurrent costs and initial start-up costs. Importantly, these costing estimates do not include any labor costs, as stocking of vending machines was incorporated within existing team members’ roles and did not require additional staff time. Potential opportunity costs for PHVM users were not estimated.

## Results

### Implementation process

In May 2022, the first and senior authors (STA, MW) presented to the Bois Forte Reservation Tribal Council about the potential public health impact of PHVM implementation. The presentation included information about the scientific literature pertaining to PHVMs and descriptions of the types of supplies that could be dispensed. The first author (STA) also shared information about his evaluation of PHVM implementation in Clark County (NV; US) that found significant reductions in overdose fatalities following launch [26]. Prior to presenting to the Reservation Tribal Council, the research team also drafted a Resolution authorizing the implementation of PHVMs on the reservation lands of the Bois Forte Band of Chippewa. The Resolution was reviewed and unanimously approved at the conclusion of the presentation to the Reservation Tribal Council (Resolution No. 101–2022). The team then collaborated with the Our Stories Matter project Community Research Council to determine the most appropriate way to implement the PHVMs and what items should be stocked in them. After identifying what items to stock in the machines, supplies were ordered using a combination of endowment funds and monies from an overdose response initiative.

In addition, our team engaged local community members, including the Reservation Tribal Council, Tribal business managers, and health and human service representatives, in discussions about where to install the PHVMs to make them most accessible and operable (e.g., indoor locations given harsh Minnesota climates). We ultimately installed one PHVM at the Tribally operated casino. Specifically, the machine was installed in the age-restricted gaming area adjacent to slot machines. A second machine was installed at a Tribally operated laundromat. Notably, each PHVM was installed at each of the two major geographic sectors of the reservation. Discussions also led to a consensus that the PHVMs should operate in ways that parallel best practices for syringe services programs and be as low-threshold as possible. To achieve this aim, PHVM access was strictly anonymous, and people were not required to register or show identification to obtain access to the machines. Further, dispensation was “needs-based” in that no limits on the volume of supplies dispensed were instituted. In doing so, people were able to obtain as many of the supplies as they required. After identifying where and how the machines would operate, our team engaged leadership at each venue to support PHVM installation.

### Community-level willingness

Prior to installation, the Our Stories Matter project in part assessed community-level willingness to use the PHVMs among a sample of people who had previously used drugs (*N* = 227) that were recruited on reservation lands. Most survey participants were women (57%), heterosexual (89%), single (53%), and had a high school education or more (88%) (Table 1). The average age of participants was 45.3 (Standard Deviation: 14.7). In the past 6 months, 16% of participants witnessed a non-fatal overdose and 6% of participants witnessed a fatal overdose. About one quarter (24%) of participants had used drugs other than cannabis in the past 6 months. Overall, 56% of the sample was willing to use a PHVM, suggesting such an intervention would be acceptable to many community members. In the bivariate analyses, age and witnessing overdoses were significantly associated with willingness to use a PHVM. On average, participants who were willing to use a PHVM were younger (M = 43) than participants who were not (M = 48, *p* = 0.011). Participants who had witnessed both non-fatal (21% vs. 8%, *p* = 0.006) and fatal overdoses (9% vs. 2%, *p* = 0.031) were more likely to report willingness to use a PHVM.

**Table 1:** Survey sample characteristics and correlates of willingness to use public health vending machines

	Total *n* = 227	Willing to use a PHVM		
		Yes (*n* = 126; 55.5%)	No (*n* = 101; 44.5%)	*p*
**Sociodemographics**				
Age, M (SD)	45.3 (14.7)	43.0 (13.7)	48.0 (15.4)	**0.011**
Gender				
Men	91 (40.1)	51 (40.5)	40 (39.6)	0.958
Women	130 (57.3)	72 (57.1)	58 (57.4)	
Other	6 (2.6)	3 (2.4)	3 (3.0)	
Sexual Minority Status	25 (11.0)	14 (11.1)	11 (10.9)	0.958
Education				
Less than High School	28 (12.3)	18 (14.3)	10 (9.9)	0.144
High school equivalent	92 (40.5)	44 (34.9)	48 (47.5)	
Some college or more	107 (47.1)	64 (50.8)	43 (42.6)	
Self-Identified Homelessness	19 (8.4)	13 (10.3)	6 (5.9)	0.237
Health Insurance	208 (91.6)	114 (90.5)	94 (93.1)	0.483
Relationship Status				
In a Relationship	106 (46.7)	63 (50.0)	43 (42.6)	0.265
Single	121 (53.3)	63 (50.0)	58 (75.43)	
Employment				
Not working	66 (29.1)	32 (25.4)	34 (33.7)	0.107
Full time	119 (52.4)	65 (51.6)	54 (53.5)	
Part time/Odd jobs	42 (18.5)	29 (23.0)	13 (12.9)	
Any Hunger, past 6 months	65 (28.6)	41 (32.5)	24 (23.8)	0.146
**Witnessing Overdoses in the past 6 months**				
Witnessed Any Non-fatal Overdoses^#^	35 (15.5)	27 (21.4)	8 (8.0)	**0.006**
Witnessed Any Fatal Overdoses^#^	13 (5.8)	11 (8.7)	2 (2.0)	**0.031**
**Indigenous Values**				
Importance of Indigenous Spirituality				
Not at all/Not too Important	20 (8.8)	7 (5.6)	13 (12.9)	0.053
Somewhat/Very Important	207 (91.2)	119 (94.4)	88 (87.1)	
Cultural Identity Scale Score, M (SD) #	18.2 (3.3)	18.1 (2.9)	18.2 (3.6)	0.936
**Used Any Drugs**,** past 6 months**	55 (24.2)	36 (28.6)	19 (18.8)	0.088
**Drug Use Characteristics (** ***n*** ** = 55)**				
Smoked Drugs	21 (38.1%)	17 (47.2)	4 (21.1)	0.082^a^
Snorted Drugs	29 (52.7%)	21 (58.3)	8 (42.1)	0.273^a^
Swallowed Drugs	36 (65.5%)	21 (58.3)	15 (79.0)	0.149^a^
Injected Drugs	14 (25.5%)	10 (27.8)	4 (21.1)	0.749^a^
Any Opioids	25 (45.5%)	16 (44.4)	9 (47.4)	1.000^a^
Any Stimulants	30 (54.6%)	22 (61.1)	8 (42.1)	0.256^a^
Any Other Drugs (except cannabis)	26 (47.3%)	18 (50.0)	8 (42.1)	0.777^a^

^#^*n* = 1 missing; ^a^ indicates use of a Fisher’s Exact test

### PHVM dispensing and utilization

The machines were installed in October 2023. In total, there were 2,534 successful dispensations at the PHVMs (1,983 at the casino, 551 at the laundromat) during the first 24 weeks of operation (Table 2). Granola bars were the most dispensed item (605 dispensed), followed by over-the-counter pain medications (311 dispensed). Key public health items dispensed through the PHVMs included: 116 COVID-19 rapid test kits, 109 nasal naloxone kits (two doses per kit for a total of 218 doses), 99 drug test strip kits, 98 menstrual supply kits, 77 wound care kits, 51 sterile injection equipment kits, 44 pregnancy test and safer sex kits, and 26 HIV self-test kits. There were 99 failed dispensations over the 24-week period, most pertaining to soft items (e.g., socks). Failed dispensations were remedied by packaging items more compactly. The PHVMs were frequently accessed on the weekends and in the evenings/at night, roughly corresponding to when each site was busier. At the casino, 44% of PHVM transactions occurred between 8:00 PM and 2:00 AM. At the laundromat, 54% of transactions occurred from 4:00 PM and 8:00 PM. Approximately two in five transactions occurred on Saturdays and Sundays.

**Table 2:** Public health vending machine dispensation from the first 24 weeks of operations

	Total	Casino	Laundromat
**Successful Transactions**	2534	1983	551
**Items Dispensed**			
Granola and Protein Bars	605	437	168
OTC Pain Relievers (e.g., Aleve, Tylenol)	311	258	53
Flashlights	132	101	31
Dental Kits	118	93	25
COVID-19 rapid tests	116	97	19
Underwear	112	81	31
Nasal Naloxone Kit	109	99	10
Drug Test Strip Kits	99	82	17
Hat/Gloves	99	79	20
Menstrual Supplies (e.g., tampons, pads)	98	85	13
Sunglasses	79	60	19
Wound Care Kit	77	54	23
Bandages	65	47	18
Socks	59	51	8
Sharps Container	58	47	11
Lip Balm	57	57	0
Glucose tablets	52	47	5
Sterile Injection Equipment Kits	51	48	3
Hand Sanitizer	46	34	12
Pregnancy Test & Condom Kit	44	31	13
Protein Drinks	42	25	17
Bottle of Water	39	32	7
Face Masks	28	13	15
HIV Self-test Kits	26	17	9
Sterile Cooker Kit	6	3	3
Sunscreen	6	5	1
**Percent of Transactions by Day of Week**			
Monday	13.4%	14.9%	8.0%
Tuesday	13.3%	12.1%	17.8%
Wednesday	11.6%	9.2%	20.1%
Thursday	11.4%	10.6%	14.2%
Friday	9.7%	9.8%	9.3%
Saturday	22.1%	22.3%	21.4%
Sunday	18.5%	21.1%	9.3%
**Percent of Transaction by Hour of Day**			
12 am − 2am	12.3%	16.0%	0.0%
2 am − 4 am	3.3%	4.3%	0.0%
4 am − 6 am	4.2%	5.5%	0.0%
6 am − 8 am	4.2%	4.8%	2.4%
8 am − 10 am	7.3%	8.0%	5.1%
10 am − 12 pm	6.1%	5.4%	8.4%
12 pm − 2 pm	8.2%	8.4%	7.6%
2 pm − 4 pm	7.2%	5.2%	13.5%
4 pm − 6 pm	9.8%	6.1%	22.0%
6pm − 8 pm	13.4%	8.2%	30.6%
8 pm − 10 pm	11.8%	12.3%	10.3%
10 pm − 12 am	12.2%	15.9%	0.2%

### PHVM implementation costs

**Fig. 1:**
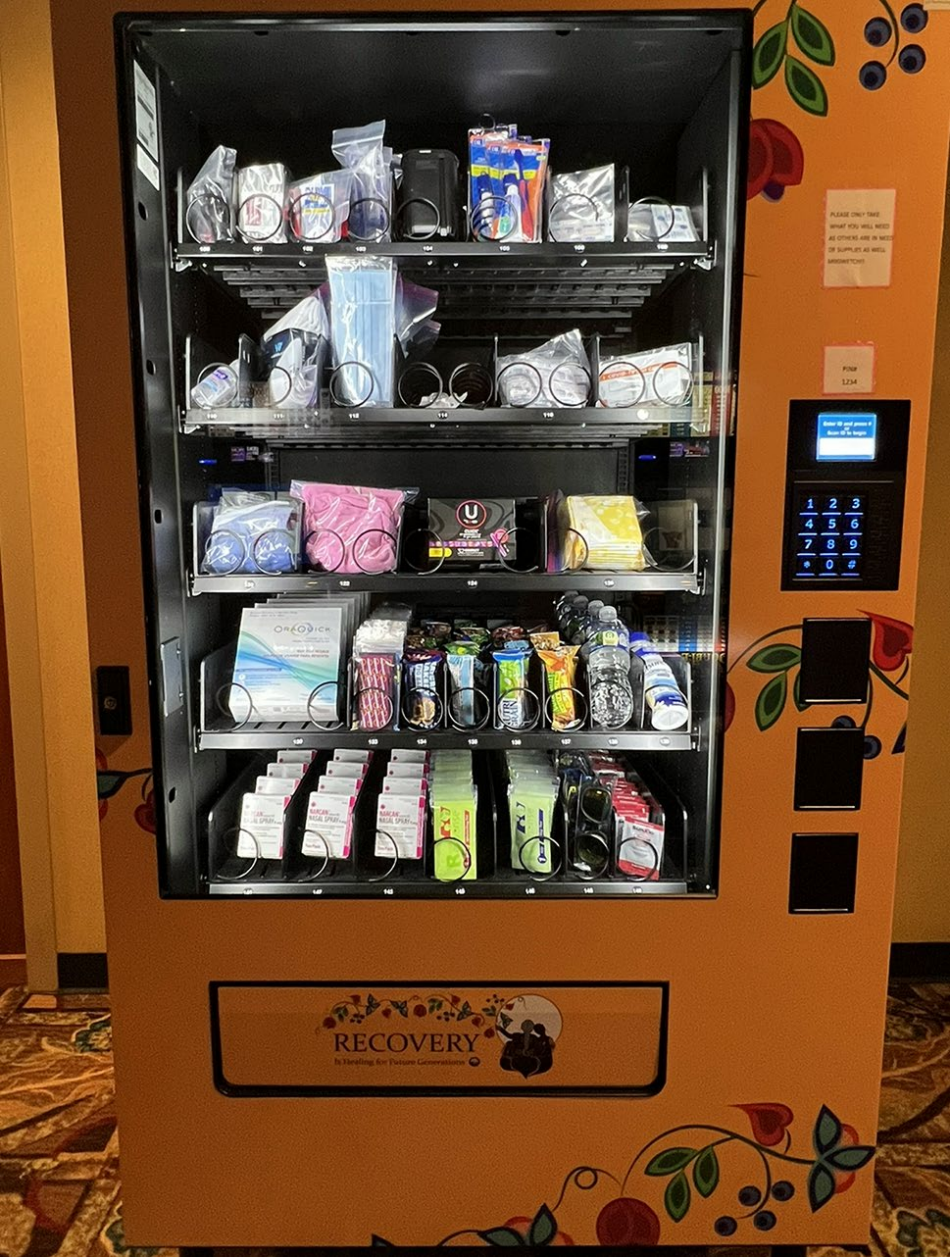
Installed PHVM

Start-up implementation costs are summarized in Table 3. In total, these expenses totaled $20,140 and include PHVM purchase, freight (e.g., shipping), and custom wraps on each machine. The custom wraps were designed by a local artist and featured Ojibwe art (e.g., braids, leaves, and flower motifs) that conveyed healing through connection and recovery for future generations (Fig. 1). We also included the annual software fee as it was required for the machines to track the vending dispensation data. In addition to the start-up costs, there were also recurrent PHVM stock expenses (Table 4). Naloxone kits were the highest cost individual item dispensed ($47.50 per kit), followed by HIV self-test kits ($29.50 per kit). Most items had small variations in prices across the study period. The total estimated recurrent stock costs during the initial 24-week period were $10,266.75. In total, the PHVM initiative cost $30,406.75 during its initial 24-week implementation period ($20,140 in start-up implementation costs and $10,266.75 in recurrent stock costs). Extrapolating these costs to a 52-week period (Table 5), we estimate that the PHVM initiative cost approximately $42,384.63 (lower bound: $40,413.26; upper bound: $45,195.53) with $20,140 in start-up implementation costs and $22,244.63 in recurrent stock costs (lower bound: $20,273.26; upper bound: $25,055.53).

**Table 3:** Public health vending machine Start-Up implementation costs

	Units	PPU	Total Item Cost
Implementation Costs (one-time)			
Vending Unit	2	$ 7,970.00	$ 15,940.00
Freight Charge	2	$ 750.00	$ 1,500.00
Custom Machine Graphics	2	$ 750.00	$ 1,500.00
Software (annual fee)	2	$ 600.00	$ 1,200.00
		**Total Implementation Costs**	$20,140.00

**Table 4:** Public health vending machine recurrent stock costs during initial 24-weeks of operations

Variable Stock	Units	Avg. PPU	PPU Range	Avg. Item Cost	Lower Bound	Upper Bound
Granola and Protein bars	605	$ 0.81	0.62–1.28	$ 490.05	$ 375.10	$ 774.40
OTC Pain Reliever (e.g., Aleve, Tylenol)	311	$ 0.29	0.06–0.42	$ 90.19	$ 18.66	$ 130.62
Flashlights	132	$ 1.31	1.17–1.40	$ 172.92	$ 154.44	$ 184.80
Dental Kits	118	$ 1.78	1.74–1.89	$ 210.04	$ 205.32	$ 223.02
COVID-19 Rapid Test	116	$ 6.56	6.29–6.67	$ 760.96	$ 729.64	$ 773.72
Underwear	112	$ 4.17	1.70–6.50	$ 467.04	$ 190.40	$ 728.00
Nasal Naloxone Kit	109	$ 47.50	--	$ 5,177.50	$ 5,177.50	$ 5,177.50
Drug Test Strip Kit	99	$ 4.00	--	$ 396.00	$ 396.00	$ 396.00
Hat/Gloves	99	$ 2.42	--	$ 239.58	$ 239.58	$ 239.58
Menstrual Supplies (e.g., tampons, pads)	98	$ 5.08	3.08–7.04	$ 497.84	$ 301.84	$ 689.92
Sunglasses	79	$ 0.75	0.69–1.80	$ 59.25	$ 54.51	$ 142.20
Wound Care Kit	77	$ 2.09	--	$ 160.93	$ 160.93	$ 160.93
Bandages	65	$ 0.30	0.20–1.20	$ 19.50	$ 13.00	$ 78.00
Socks	59	$ 2.30	2.22–2.64	$ 135.70	$ 130.98	$ 155.76
Sharps Container	58	$ 2.56	2.36–2.76	$ 148.48	$ 136.88	$ 160.08
Lip Balm	57	$ 0.79	0.32–1.29	$ 45.03	$ 18.24	$ 73.53
Glucose tablets	52	$ 3.34	2.92–4.17	$ 173.68	$ 151.84	$ 216.84
Sterile Injection Equipment Kit	51	$ 0.65	0.64–0.66	$ 33.15	$ 32.64	$ 33.66
Hand Sanitizer	46	$ 1.66	1.00–3.67	$ 76.36	$ 46.00	$ 168.82
Pregnancy Test & Condom Kit	44	$ 1.09	0.67–1.89	$ 47.96	$ 29.48	$ 83.16
Protein Drinks	42	$ 1.74	1.65–1.78	$ 73.08	$ 69.30	$ 74.76
Bottle of Water	39	$ 0.33	--	$ 12.87	$ 12.87	$ 12.87
Face Masks	28	$ 0.27	0.26–0.28	$ 7.56	$ 7.28	$ 7.84
HIV Self-test Kits	26	$ 29.50	27.00–33.56	$ 767.00	$ 702.00	$ 872.56
Sterile Cooker Kit	6	$ 0.18	0.17–0.19	$ 1.08	$ 1.02	$ 1.14
Sunscreen	6	$ 0.50	0.24–0.73	$ 3.00	$ 1.44	$ 4.38
	**Estimated 24-week material costs**			$10,266.75	$9,356.89	$11,564.09

**Table 5:** Total estimated One-Year operational costs of PHVM initiative by recurrent stock costs

Recurrent Stock Costs	Ave. Item Cost $22,244.63	Lower Bound $20,273.26	Upper Bound $25,055.53
Start-Up Implementation Costs ($20,140)	$42,384.63	$40,413.26	$45,195.53

## Discussion

This research builds on existing scientific literature by describing each step of PHVM implementation on the reservation lands of the Bois Forte Band of Chippewa. As indicated by survey data collected prior to PHVM implementation, there was a high level of willingness to use the machines among people who use drugs. These findings were corroborated by the volume of supply dispensation during the first 24 weeks of operations. Data generated from this study indicates PHVMs are an evidence-based intervention to increase access to essential, lifesaving, and health-promoting public health resources. Communities should consider PHVM implementation in combination with other evidence-based interventions and strategies for mitigating public health inequities. This study makes a unique contribution to public health given that the Bois Forte Band of Chippewa was one of the first tribes in the US to implement PHVMs [31–33]. 

This study documents the value of communities translating research to action at each stage of intervention implementation. At the onset of this study, the Bois Forte Reservation Tribal Council learned about the public health benefits of PHVM implementation in other communities and issued a unanimous Resolution authorizing implementation on Reservation lands. This action is noteworthy not only because of the expediency with which the Reservation Tribal Council acted, but also because they did so when there was no known precedent for PHVM implementation in other Native communities and only a limited number of PHVM implementation contexts in other jurisdictions in the US. This research used survey data to understand community-level needs for PHVM operations prior to implementation. These data directly informed how we understood their potential impact among people who used drugs. Finally, the Community Research Council and academic collaborators used dispensation data in real-time to inform what was stocked in the machines. Taken together, this initiative demonstrates that community-based participatory approaches anchored in using research evidence to tailor implementation processes to culture and context are critical for the success of public health interventions in Indigenous communities.

We found that willingness to use a PHVM was significantly associated with persons having witnessed overdoses. This finding suggests that people who use drugs may benefit greatly from PHVM implementation, particularly machines that dispense the overdose reversal medication naloxone and drug checking supplies. This finding aligns with existing scientific evidence; for example, a 2022 study found that there was an approximate 15% reduction in overdose mortality in the year following the launch of naloxone dispensation at PHVMs in Clark County, NV (US) [26]. Given worsening trends in overdose morbidity and mortality, ensuring people who use drugs and their network affiliates have consistent, low-threshold access to overdose prevention resources is a priority. Additional research should be conducted to better understand where to place PHVMs to maximize their utilization among people who use drugs.

The initial 24-week period of PHVM utilization data suggests the intervention helped address community-level needs for an array of public health supplies. For example, a large volume of public health test kits (e.g., COVID-19, HIV, pregnancy) were dispensed along with preventative supplies (e.g., wound care kits, bandages, drug checking supplies). The PHVMs were also successful at dispensing clothing items, including socks, hats, and gloves. In total, there were more than 2,500 dispensation events at the PHVMs during the first 24-weeks of operations. Given the diversity of items made available at the machines, these data suggest PHVM implementation could be an effective strategy to improve a range of community-level health conditions. For instance, the PHVMs at the Bois Forte Band of Chippewa dispensed items pertinent to diabetes (i.e., glucose tablets), HIV prevention (i.e., sterile injection equipment, self-test kits, condoms), oral hygiene (i.e., dental kits), substance use (i.e., harm reduction supplies), and hunger (i.e., snacks). Notably, the items dispensed at PHVMs can be easily customized to local needs and seasonality, underscoring their potential as a readily adaptable intervention across diverse contexts.

Indigenous communities throughout the US experience significant health consequences stemming from colonization, intergenerational traumas, and loss of ancestral lands and culture [7–9]. In many cases, these events created a justifiable mistrust of interventions developed outside of Native communities. The Bois Forte Band of Chippewa should be commended for its proactive approach toward PHVM implementation, including how the machines were tailored to fit local culture and context. Prior to installation, the machines were wrapped in artwork from a local artist. This artwork did more than visually set the PHVMs apart from standard snack machines; the artwork served as a symbol of cultural pride and demonstrated that the PHVMs were designed and operated by and for Indigenous Peoples. Additionally, the Community Research Council ensured the PHVMs operated in a way that aligned with underlying community expectations, attitudes, beliefs, and culture. Most notably, the PHVMs also afforded anonymous, “needs-based” supply access. These attributes parallel evidence-based best practices for syringe services programs and were a clear contributor to success as persons with any number of conditions could access the machines without judgement or fear of identification. The success and ease with which PHVMs were implemented at the Bois Forte Band of Chippewa should serve as a model for other communities to follow and an example of successful collaboration between academic and community partners.

The costs associated with PHVM implementation were modest relative to the potential public health impact they may have offered to the local community. In other words, the diversity of supplies and the volumes dispensed suggest the PHVMs were an important investment in community health. Notably, the PHVMs were not designed to replace other interventions (e.g., community health workers), but rather enhance access to health-promoting supplies. The PHVMs were viewed as an enhancement to the existing healthcare infrastructure at the Bois Forte Band of Chippewa. Nevertheless, communities should develop sustainability plans prior to and throughout their PHVM implementation processes as some items are costly. For instance, naloxone and HIV self-test kit dispensation requires significant investment if the items are widely distributed. However, the benefits of preventing overdose and HIV transmission justify these expenditures. For example, lifetime HIV treatment costs for a single person exceed $420,000– nearly ten times the estimated costs of PHVM operations at the Bois Forte Band of Chippewa for a single year [43]. Ensuring sustainable PHVM operations requires communities to act proactively and seek funding from local, state, Federal, and private funders.

This research has limitations that should be acknowledged. Our study reflects the PHVM implementation processes and utilization at the Bois Forte Band of Chippewa. Given that the PHVMs were tailored to local needs, other communities may have different experiences implementing machines. Further, implementation approaches may differ based on whether an academic partner is involved. Another limitation is that we only surveyed people who had histories of substance use. As a result, our survey data likely undercount the true willingness to use a PHVM as the machines offered supplies relevant to numerous health conditions. Additionally, our costing analyses were limited to the first 24 weeks of PHVM operations. Price variability of items may limit cost predictability in other implementation settings. For example, nasal naloxone prices were significantly lower at the time of publication than they were during our initial purchase, while COVID-19 test pricing fluctuates with demand. Our cost estimates did not include labor or user opportunity costs, though we anticipate that both costs would have been relatively small. Finally, this research did not evaluate potential associations between PHVM implementation and shifts in disease morbidity or mortality as these types of studies would require robust surveillance data that were not available. Despite these limitations, this research makes a meaningful contribution to the public health literature by describing PHVM implementation on the Bois Forte Band of Chippewa Reservation lands. To the best of our knowledge, this is the first study of PHVM implementation processes on American Indian Reservation lands.

In conclusion, this research describes how PHVMs were implemented on the Bois Forte Band of Chippewa Reservation lands in Minnesota (US). The Bois Forte Band took swift action upon learning about the potential public health benefits of PHVMs and immediately authorized their implementation, catalyzing a collaborative effort between local community members and academic partners. Thousands of life-sustaining supplies were dispensed at the PHVMs, supporting the prevention and treatment needs of persons with many conditions. PHVM implementation is an evidence-based strategy communities should include as an additional component of holistic public health initiatives.

## Data Availability

The datasets generated and analyzed during the current study are not publicly available as determined by the data sovereignty agreement with the collaborating Tribal Nation. They are not publicly available due to privacy concerns.

## Abbreviations

PHVMs: Public health vending machines
HIV: Human immunodeficiency virus
US: United States
CRC: Community Research Council
OTC: Over the counter
CBPR: Community Based Participatory Research

## References

[1] Friedman JR, Hansen H. Evaluation of increases in drug overdose mortality rates in the US by race and ethnicity before and during the COVID-19 pandemic. JAMA Psychiatry. 2022;79(4):379–81. 35234815 10.1001/jamapsychiatry.2022.0004 PMC8892360

[2] Qeadan F, et al. Epidemiological trends in opioid-only and opioid/polysubstance-related death rates among American Indian/Alaska native populations from 1999 to 2019: a retrospective longitudinal ecological study. BMJ Open. 2022;12(5):e053686. 35501103 10.1136/bmjopen-2021-053686 PMC9109082

[3] Schuler MS, Schell TL, Wong EC. Racial/ethnic differences in prescription opioid misuse and heroin use among a National sample, 1999–2018. Drug Alcohol Depend. 2021;221:108588. 33639569 10.1016/j.drugalcdep.2021.108588 PMC8026521

[4] Centers for Disease Control and Prevention. HIV in the United States by Race/Ethnicity: HIV Risk Behaviors. Available at: https://www.cdc.gov/hiv/data-research/facts-stats/race-ethnicity.html

[5] Spencer MR, Miniño AM, Warner M. Drug Overdose Deaths in the United States, 2001-2021. NCHS Data Brief. 2022;(457):1–8. PMID: 36598401. https://www.cdc.gov/nchs/products/databriefs/db457.htm 36598401

[6] Bauer C, et al. Trends in fatal Opioid-Related overdose in American Indian and Alaska native Communities,1999–2021. Am J Prev Med. 2024;66(6):927–35. 38311190 10.1016/j.amepre.2024.01.019 PMC11843516

[7] King M, Smith A, Gracey M. Indigenous health part 2: the underlying causes of the health gap. Lancet. 2009;374(9683):76–85. 19577696 10.1016/S0140-6736(09)60827-8

[8] Gameon JA, Skewes MC. Historical trauma and substance use among American Indian people with current substance use problems. Psychol Addict Behav. 2021;35(3):295–309. 33829816 10.1037/adb0000729 PMC8084991

[9] Pokhrel P, Herzog TA. Historical trauma and substance use among native Hawaiian college students. Am J Health Behav. 2014;38(3):420–9. 24636038 10.5993/AJHB.38.3.11 PMC4877175

[10] Kelley A, et al. Tribally-led mobile outreach: improving access to harm reduction services in one rural reservation community. Front Public Health. 2024;12:1383729. 38818437 10.3389/fpubh.2024.1383729 PMC11137169

[11] Kebec P, Remacle C, Conley A, Akerman S, Tocheterman A. Expanding the circle of care a practical guide to syringe services for tribal and rural communities. Available at: https://static1.squarespace.com/static/61b2437f05011b1f37605c04/t/6228de5e5f55652e8464ccf0/1646845536975/CircleofCare+web%281%29+%281%29.pdf

[12] National Harm Reduction Coalition. Native Harm Reduction Toolkit. Available at: https://harmreduction.org/native-toolkit/

[13] National Harm Reduction Coalition. Principles of Harm Reduction. Available at: https://harmreduction.org/about-us/principles-of-harm-reduction/

[14] Harm Reduction International. What is harm reduction? Available at: https://hri.global/what-is-harm-reduction/

[15] Centers for Disease Control and Prevention. Syringe Services Programs. Available at: https://www.cdc.gov/syringe-services-programs/php/index.html

[16] Ruiz MS, et al. Using interrupted time series analysis to measure the impact of legalized syringe exchange on HIV diagnoses in Baltimore and Philadelphia. J Acquir Immune Defic Syndr. 2019;82(Suppl 2):S148–54. 31658203 10.1097/QAI.0000000000002176 PMC6820712

[17] Ruiz MS, O’Rourke A, Allen ST. Impact evaluation of a policy intervention for HIV prevention in Washington, DC. AIDS Behav. 2016;20(1):22–8. 26336945 10.1007/s10461-015-1143-6 PMC4715855

[18] McAuley A, Aucott L, Matheson C. Exploring the life-saving potential of Naloxone: a systematic review and descriptive meta-analysis of take home Naloxone (THN) programmes for opioid users. Int J Drug Policy. 2015;26(12):1183–8. 26508033 10.1016/j.drugpo.2015.09.011

[19] McDonald R, Campbell ND, Strang J. Twenty years of take-home Naloxone for the prevention of overdose deaths from heroin and other opioids—conception and maturation. Drug Alcohol Depend. 2017;178:176–87. 28654870 10.1016/j.drugalcdep.2017.05.001

[20] McDonald R, Strang J. Are take-home Naloxone programmes effective? Systematic review utilizing application of the Bradford hill criteria. Addiction. 2016;111(7):1177–87. 27028542 10.1111/add.13326 PMC5071734

[21] Strang J, et al. Take-home Naloxone for the emergency interim management of opioid overdose: the public health application of an emergency medicine. Drugs. 2019;79:1395–418. 31352603 10.1007/s40265-019-01154-5 PMC6728289

[22] Keane C, Egan JE, Hawk M. Effects of Naloxone distribution to likely bystanders: results of an agent-based model. Int J Drug Policy. 2018;55:61–9. 29524734 10.1016/j.drugpo.2018.02.008

[23] Weiner J, Murphy SM, Behrends C. Expanding access to Naloxone: a review of distribution strategies. Issue Brief. 2019;23:132.

[24] Indigenous Peoples Task Force. About IPTF. Available at: https://indigenouspeoplestf.org/about-iptf/

[25] Russell E, et al. A scoping review of implementation considerations for harm reduction vending machines. Harm Reduct J. 2023;20(1):33. 36927354 10.1186/s12954-023-00765-2 PMC10018614

[26] Allen ST, et al. Evaluating the impact of Naloxone dispensation at public health vending machines in Clark County, Nevada. Ann Med. 2022;54(1):2692–700. 36168975 10.1080/07853890.2022.2121418 PMC9542801

[27] Islam MM, Conigrave KM. Syringe vending machines as a form of needle syringe programme: advantages and disadvantages. J Subst Use. 2007;12(3):203–12.

[28] Obadia Y, et al. Syringe vending machines for injection drug users: an experiment in Marseille, France. Am J Public Health. 1999;89(12):1852–4. 10589315 10.2105/ajph.89.12.1852 PMC1509009

[29] Islam M, Wodak A, Conigrave KM. The effectiveness and safety of syringe vending machines as a component of needle syringe programmes in community settings. Int J Drug Policy. 2008;19(6):436–41. 17766100 10.1016/j.drugpo.2007.07.006

[30] McDonald D. The evaluation of a trial of syringe vending machines in Canberra, Australia. Int J Drug Policy. 2009;20(4):336–9. 18790622 10.1016/j.drugpo.2008.06.004

[31] Lee L. Bois Forte becomes first tribal nation in MN to introduce Public Health Vending Machines. 2023. https://www.northernnewsnow.com/2023/12/08/bois-forte-becomes-first-tribal-nation-mn-introduce-public-health-vending-machines/

[32] Wild E. Bois Forte tribe takes on overdoses, infectious diseases with vending solutions. Available at: https://www.northernnewsnow.com/2023/12/08/bois-forte-becomes-first-tribal-nation-mn-introduce-public-health-vending-machines/.

[33] Anderson BK. New health care vending machines go live Friday. Available at: https://www.timberjay.com/stories/new-health-care-vending-machines-go-live-friday,20700

[34] Bois Forte Band of Chippewa. A Brief Summary of Bois Forte History. Available at: https://boisforte.com/about/history/

[35] United States CensusBureau. Bois Forte Reservation and Off-Reservation Trust Land, MN. Available at: https://censusreporter.org/profiles/25000US0335-bois-forte-reservation-and-off-reservation-trust-land/

[36] NCAI Policy Research Center and MSU Center for Native Health Partnerships. (2012). ‘Walk softly and listen carefully’: Building research relationships with tribal communities. Washington, DC, and Bozeman, MT: Authors.

[37] Israel BA, et al. Review of community-based research: assessing partnership approaches to improve public health. Annu Rev Public Health. 1998;19:173–202. 9611617 10.1146/annurev.publhealth.19.1.173

[38] Smith LT. Decolonizing methodologies: research and Indigenous peoples. Otago University; 2012.

[39] Becker, A., Israel, B.A., Gustat, J., Reyes, A.G., Allen, A.J., & Becker, A.B. (2012). Strategies and Techniques for Effective Group Process in Community-Based Participatory Research Partnerships.

[40] BlackDeer AA. *Decolonizing Data. Available at: https://pressbooks.pub/indigenizingclimateaction/chapter/decolonizing-data-the-movement-for-indigenous-data-sovereignty/*

[41] O’Connor J, et al. Decolonizing data visualization: A history and future of Indigenous data visualization. J MultiDisciplinary Evaluation. 2023;19(44):62–79.

[42] Leach CW, et al. Group-level self-definition and self-investment: a hierarchical (multicomponent) model of in-group identification. J Personal Soc Psychol. 2008;95(1):144. 10.1037/0022-3514.95.1.144 18605857

[43] Bingham A, et al. Estimated lifetime HIV-Related medical costs in the united States. Sex Transm Dis. 2021;48(4):299–304. 33492100 10.1097/OLQ.0000000000001366

